# Amino Acid Catabolism in Alzheimer's Disease Brain: Friend or Foe?

**DOI:** 10.1155/2017/5472792

**Published:** 2017-02-05

**Authors:** Jeddidiah W. D. Griffin, Patrick C. Bradshaw

**Affiliations:** Department of Biomedical Sciences, East Tennessee State University College of Medicine, Johnson City, TN 37614, USA

## Abstract

There is a dire need to discover new targets for Alzheimer's disease (AD) drug development. Decreased neuronal glucose metabolism that occurs in AD brain could play a central role in disease progression. Little is known about the compensatory neuronal changes that occur to attempt to maintain energy homeostasis. In this review using the PubMed literature database, we summarize evidence that amino acid oxidation can temporarily compensate for the decreased glucose metabolism, but eventually altered amino acid and amino acid catabolite levels likely lead to toxicities contributing to AD progression. Because amino acids are involved in so many cellular metabolic and signaling pathways, the effects of altered amino acid metabolism in AD brain are far-reaching. Possible pathological results from changes in the levels of several important amino acids are discussed. Urea cycle function may be induced in endothelial cells of AD patient brains, possibly to remove excess ammonia produced from increased amino acid catabolism. Studying AD from a metabolic perspective provides new insights into AD pathogenesis and may lead to the discovery of dietary metabolite supplements that can partially compensate for alterations of enzymatic function to delay AD or alleviate some of the suffering caused by the disease.

## 1. Introduction

There are currently about 24 million cases of Alzheimer's disease (AD) worldwide, and that number is expected to continue to increase for at least the next few decades as better treatments for other diseases such as heart disease and cancer extend average human longevity [1]. In addition to human suffering, Alzheimer's disease and other dementias cost the United States about $172 billion in 2010 [2]. To date, a majority of research has focused on the amyloid cascade hypothesis that emphasizes the role of amyloid-*β* protein aggregation in the pathogenesis of AD. However, growing evidence suggests that the amyloid cascade hypothesis does not encapsulate the complex symptomology of AD [3]. Two decades of researching the amyloid cascade hypothesis have not yielded the treatments that were predicted in the early 1990s. The other major histological hallmark of AD brain in addition to amyloid plaques is the neurofibrillary tangle pathology resulting from hyperphosphorylation and aggregation of tau protein [3]. It is possible that tau-based therapies will not fare better in clinical trials than amyloid-based therapies. Another promising alternative is to view Alzheimer's disease as a metabolic disease in attempt to shed novel insight into its etiology. In this regard, it is known that neurons in AD brain show large deficits in glucose metabolism, so alternative energy sources may help to prevent the neuronal death characteristic of the disease. Treating AD as a metabolic disorder would lead to further research into dietary supplementation of metabolites and enzyme cofactors. One such strategy is to supplement with factors that are depleted in AD brain, while another strategy is to supplement with metabolites that can be oxidized to provide energy for neurons. Neurons lack the enzymes for beta-oxidation of fatty acids, but other possible neuronal energy sources include amino acids, ketone bodies, citric acid cycle intermediates, pyruvate, and lactate. Because many recent metabolomics investigations have shown large changes in the levels of several amino acids in AD brain and plasma, it is important to consider whether changes in amino acid metabolism are a driving force for AD progression.

Amino acids in the form of proteins are a large part of the human diet. The recommended daily allowance of protein is 0.8 grams per kilogram body mass [4]; in an average adult this amounts to roughly 71 grams of protein per day. This high protein consumption dictates that amino acids will be present at levels that far exceed their requirements as the building blocks for protein synthesis, and most of the protein consumed will be broken down for energy generation. Processing these amino acids for energy generation requires the disposal of nitrogenous waste, a process carried out mostly in the liver and small intestine by the urea cycle. Disrupted amino acid and nitrogen metabolism is associated with neurological defects and in some cases dementia [5–9]. In addition to these primary routes of amino acid usage, amino acids and their metabolic derivatives are involved to a lesser extent in cell signaling and in many diverse metabolic pathways.

## 2. Amino Acid Metabolism in the AD Brain

Many studies have shown altered amino acid levels in serum and brain in AD patients or in AD model mice, but whether or not these changes contribute to disease pathogenesis is not yet known. Because glutamate is an excitatory neurotransmitter and one of its metabolites, gamma-aminobutyric acid (GABA), is an inhibitory neurotransmitter [9], changes in glutamate metabolism in AD brain could greatly affect neural functioning. GABA can also be synthesized from arginine in astrocytes and increased GABA levels synthesized through this pathway have been shown to play a role in cognitive dysfunction in an AD mouse model [31]. Changes in the levels of enzymes involved in amino acid metabolism have also been observed in AD brain [32], further suggesting a role for metabolic dysregulation in AD pathogenesis. For example, alterations in the levels of glutamine synthetase and urea cycle enzymes and intermediates have been observed in AD brain. This is of concern because nitrogenous waste in the form of ammonia from amino acid catabolism has adverse effects on neural cells if not properly cleared from the brain.

A healthy individual is able to process the excess amino acids consumed into other useful metabolites or oxidize them for energy production. To use them as fuel, the carbon skeletons are oxidized in the citric acid cycle to produce carbon dioxide, and the excess nitrogen is disposed of as the relatively nontoxic nitrogenous waste product urea. However, when neurons cannot catabolize glucose efficiently, such as during AD, they may become reliant upon amino acid oxidation for energy production. If neuronal amino acids become depleted or if the machinery used to metabolize amino acids becomes dysregulated, the neurons may die, contributing to disease progression. However, even if amino acid oxidation is able to maintain neuronal energy levels, the increased amounts of ammonia released during amino acid catabolism could lead to neuronal cell death because all of the enzymes of the urea cycle needed to detoxify ammonia are not present in neurons or glia. Instead, astrocytes express high levels of the glutamine synthetase enzyme to sequester ammonia into glutamine, which is then released from the brain. Due to the 40-fold lower expression of glutamine synthetase in neurons, they are not equipped to detoxify the excess ammonia.

## 3. Amino Acid Level Changes in Control, MCI, and AD Populations

Identifying amino acid changes in patients with mild cognitive impairment (MCI) is important as these changes may be upstream changes leading to the onset of AD, and the downstream changes that occur during AD may be a result of the body adapting to the insults that occurred during MCI. In one report, a metabolomics analysis was performed on the cerebrospinal fluid (CSF) of patients with MCI, AD, or healthy aged-matched controls. Results showed that the metabolites dimethylarginine, arginine, valine, proline, serine, histidine, choline, creatine, carnitine, and suberylglycine were possible disease progression biomarkers [33]. Another group studying potential CSF biomarkers for AD concluded that changes in methionine, tryptophan, tyrosine, and purine metabolism pathways occurred in both MCI and AD subjects. Methionine levels increased in MCI while tryptophan levels decreased [34]. Levels of the tripeptide glutathione also decreased in AD. One study found increased cysteine levels in CSF from AD subjects [28], while another one identified altered tryptophan and phenylalanine levels in plasma from both MCI and AD subjects compared to controls; tryptophan levels were also distinct when comparing MCI to AD subjects [35]. A further metabolomics study of plasma found altered arginine metabolism and polyamine metabolism in MCI and AD subjects [36]. Another study found that glycine and valine levels were altered in AD plasma [37], but the authors warned that plasma amino acid levels show large variability depending upon the amount of fasting the subjects had undergone prior to donating blood [38] and that phospholipids may be more reliable plasma biomarkers.

A comprehensive metabolomics study of both plasma and CSF from control, MCI, and AD subjects found that tryptophan and arginine metabolism were altered in both CSF and plasma from MCI subjects [39]. Lysine metabolism was decreased in the CSF but not plasma from the MCI subjects. This study also found increased methionine levels in the CSF of MCI subjects. Methionine, histidine, and lysine levels were increased in AD plasma. The pathways affected in both AD CSF and plasma included beta-alanine, aspartate and asparagine, alanine, cysteine, methionine, methionine-cysteine-glutamate, and arginine and lysine metabolism. Phenylalanine, lysine, and leucine were three of six metabolites in plasma that could be used to discriminate between the MCI subjects and controls [39]. A salivary metabolomics analysis found that taurine and several dipeptides including Ser-Ser, Phe-Pro, and Arg-Leu were decreased in abundance in MCI patients [40]. From the summation of these results one can discern that there are many alterations in amino acid metabolism in MCI and AD patients, but the results are not very consistent from study to study likely due to the different methodologies and instrumentation used.

## 4. An Overview of Select Amino Acids by Class

In MCI and AD subjects, some amino acids increased in abundance while most decreased in abundance, especially in the brain, consistent with their oxidation as a neuronal energy source. This information is summarized in Table 1. There are several possible mechanisms described in detail below through which altered metabolism of specific amino acids may lead to neural pathogenicity.

### 4.1. Branched Chain Amino Acids, mTOR, and AD

The branched chain amino acids (BCAAs) include leucine, valine, and isoleucine. BCAAs compete with the aromatic amino acids phenylalanine, tyrosine, and tryptophan for entry into the brain. Therefore, altering plasma BCAA levels can affect the levels of the neurotransmitters serotonin, dopamine, epinephrine, and norepinephrine in the brain [41]. Unlike most amino acids which are metabolized to a large extent by first pass hepatic metabolism, BCAAs are not metabolized there to a large degree, so their concentration in blood often directly reflects the level of dietary consumption. Protein restriction has been shown to decrease tau hyperphosphorylation and increase cognition in an AD mouse model [42]. A BCAA-restricted diet has been shown to induce protective metabolic effects on peripheral glucose and insulin levels in a similar manner as a protein restricted diet [43]. However, whether the neuroprotective effects of protein restriction are mediated by decreased BCAA levels is not yet known as restriction of other amino acids such as methionine can also lead to protective metabolic effects [44].

Some researchers suspect a link between increased BCAA levels and AD pathogenesis [45]. Increasing BCAA levels through dietary supplementation in rats led to a decrease in neural growth factor (NGF) in the hippocampus [46], a part of the brain involved in memory formation and known to undergo extensive neuronal loss in AD patients. Administration of the leucine metabolite *α*-ketoisocaproic acid also decreased NGF as well as brain-derived neurotrophic factor (BDNF) [11]. However, the role of BCAAs in AD is not clear-cut. The level of one of the BCAAs, valine, was found to be decreased in the plasma of AD patients [14]. Furthermore, there are several studies that link increased levels of BCAAs to indicators of increased health such as increased muscle protein synthesis [47], mitochondrial biogenesis [13], and mTOR signaling [48]. A beneficial role of mTOR signaling in AD has been hypothesized due to the fact that insulin signaling is neuroprotective [49, 50], and insulin can activate mTOR kinase through PI3K [51, 52]. mTOR activity has also been found to be neuroprotective under other experimental conditions. For example, increased mTOR activation was associated with decreased A*β* pathology in brains from the 5xFAD mouse model [53]. However, these effects may be related to the particular model or to the length of time of mTOR activation. In the short term, it appears that mTOR activation can lead to improved insulin secretion [54], whereas chronic mTORC1 activation may lead to exhaustion of *β*-cells in the pancreas [55], decreasing the levels of neuroprotective insulin.

Somewhat contrary to the findings of mTOR being neuroprotective is the finding that increased mTOR activation is frequently found in the brains of AD model mice [56] and human AD patients [57]. Increased amyloid-beta levels lead to increased mTOR activation, which increases protein translation to increase levels of tau protein, the main component of the neurofibrillary tangles pathologically found in AD neurons [58]. The increased rate of translation induced by mTOR activation may be partially responsible for the decreased levels of amino acids measured in AD brain. Increased mTOR activity can also stimulate mitochondrial electron transport chain activity [59], perhaps leading to increased mitochondrial catabolism of amino acids. The increased mTOR activity also decreases the rate of autophagy, leading to the buildup of toxic amyloid-beta peptides. Consistent with these findings, treatment of PDAPP or 3xTg-AD mice with rapamycin, an mTOR inhibitor, reduced amyloid-beta and tau levels and restored cognitive function [60, 61]. These data suggest that diets containing low protein levels or low levels of the potent mTOR activators leucine and arginine may prove beneficial for AD patients [62], although decreasing the levels of all three BCAAs together was more potent than decreasing only leucine levels on enhancing peripheral metabolism in mice [43].

Since a portion of the protective effects of mTOR inhibition by rapamycin treatment in AD model systems results from a decreased rate of translation, other therapies which decrease the rate of translation in the brain may also be therapeutic. With this in mind, decreased or unbalanced amino acid levels have also been shown to decrease the rate of translation through the general control nonderepressible 2- (GCN2-) eIF2*α* kinase pathway. GCN2 kinase senses uncharged tRNAs and then phosphorylates the translation initiation factor eIF2*α* to slow the rate of translation. As mentioned above, many amino acids, for example, BCAAs and aromatic amino acids, share the same amino acid transporter for transport across the blood-brain barrier. Therefore, supplementation with high levels of one particular amino acid may decrease the rate of transport of others into the brain to decrease their levels. This could create imbalanced amino acid levels in the brain to activate GCN2 and inhibit mTOR, decreasing global translation rates and increasing autophagy to protect AD brains. Not all mRNA transcripts show decreased translation under amino acid limitation. Some transcripts such as ATF4 show increased translation to mediate protective compensatory responses. ATF4 is a transcription factor involved in the ER stress response that has been shown to be upregulated in liver during five conditions that extended mouse longevity [63].

There are two prevalent hypotheses linking BCAAs to metabolic disease [10]. First, increased levels of BCAAs, especially leucine, may directly lead to persistent activation of mTORC1. Second, hyperactivation of BCAA catabolism can lead to increased BCAA metabolites which lead to metabolic dysfunction. For example, adding the *α*-ketoacid catabolite of leucine, *α*-ketoisocaproic acid, to rat neurons led to mitochondrial dysfunction [12]. The conflicting results of studies attempting to find correlations between BCAA levels and disease suggest a complex role for BCAA levels in metabolism that may vary depending on the model organism, disease state, and the length of time of elevated BCAA and BCAA catabolite levels.

Few studies have examined the levels of BCAAs specifically in postmortem AD brain, but it appears that increasing certain BCAAs may be beneficial for the aging brain in specific model systems. A diet high in BCAAs has even been shown to increase the mean lifespan of male mice [13]. Research into the role of BCAAs in AD is far from complete. For example, there has been little study of the effects of isoleucine or valine supplementation in AD patients or animal models. Since isoleucine and valine do not stimulate mTOR activity as potently as leucine [64] and are not broken down into neurotoxic *α*-ketoisocaproic acid, supplementation with these amino acids could provide energy for the brain without activating potentially pathogenic signaling pathways. Given the links between BCAAs, mTOR signaling, aging, and neurodegeneration, further research will likely clarify these complex interactions. Furthermore, many of the same correlations observed for BCAAs in insulin resistant individuals have also been observed for aromatic amino acids, highlighting a complementary role for both of these classes of amino acids in metabolism and disease [65].

### 4.2. Aromatic Amino Acids

Several studies have found that the levels of aromatic amino acids are altered in AD serum or brain. One research group found a decrease in all three aromatic amino acids in the serum of AD patients [14], while others reported an increase in both phenylalanine and tryptophan in the brains of AD patients [15]. Researchers using AD model rats found an increase in phenylalanine in different regions of the brain [16]. In the brain, tryptophan has two different metabolic fates: it can be metabolized into serotonin or it can enter the kynurenine pathway (KP) where it is degraded to *α*-amino-*β*-carboxymuconate-*ε*-semialdehyde (ACMS), which can be metabolized either into quinolinic acid for NAD synthesis or into 2-aminomuconate for entry into the TCA cycle. Serotonin plays a role in learning and cognition [66], but enzymes involved in the KP are upregulated in AD [18]. There is evidence that quinolinic acid (QUIN) and other metabolic intermediates in the KP pathway cause oxidative damage to the brain [18]. Increased QUIN led to a concentration-dependent increase in ROS levels in the synaptosomes of rat brains and lipid peroxidation in the hippocampus [67]. It has also been shown that QUIN can increase nitric oxide synthase activity more than threefold [19]. This enzyme produces nitric oxide, a vasorelaxing free radical. Amyloid-beta production increased the concentration of QUIN [68], linking AD more directly to oxidative damage from amino acid metabolites. Moreover, a shift in tryptophan degradation to the KP pathway diverts tryptophan from the serotonin synthesis pathway. This could deprive the AD brain of serotonin, contributing to the pathogenesis of AD. However, knowledge of aromatic amino acid metabolism and signaling in AD brain is far from complete. Dietary tryptophan restriction has been shown to extend the lifespan of rodents [69], but the mechanism has not been investigated. Acute tryptophan depletion leads to memory impairment in humans [70].

The amino acid tyrosine is important for synthesizing catecholamines, but there are only a few studies measuring tyrosine levels specifically in AD brain, although several studies have found that oral tyrosine administration improves memory and cognitive function [20]. Phenylalanine can be metabolized through the same metabolic pathways as tyrosine, but only when tyrosine levels are low [17], preventing firm conclusions regarding changes in phenylalanine levels in AD brain until changes in tyrosine levels are measured as well. As a class, aromatic amino acid metabolism is especially important for neural functioning, and more research is needed to elucidate the relevance of the changes that occur in AD.

### 4.3. Charged Amino Acids

The charged amino acids include the acidic (aspartate and glutamate) and basic (arginine, lysine, and histidine) amino acids. Each of these appears to be decreased in AD patient brain or plasma, with the possible exception of glutamate where the direction of change may depend upon the brain region assayed. Glutamate [23], histidine, and aspartate levels were decreased in serum from AD patients [14], while aspartate and glutamate levels were decreased in the temporal lobe of the cerebral cortex of AD patients [22]. Xu et al. measured a decrease in both lysine and aspartate levels in the brains from autopsied AD patients, while glutamate levels increased [15]. Glutamate's interaction with the NMDA receptor is critical for learning and memory formation [9], but glutamate excitotoxicity leads to neuronal death in AD [24, 25]. As discussed below, aspartate and glutamate also play a role in transamination reactions such as those occurring upstream of the urea cycle. As a group, the levels of charged amino acids are altered in AD, suggesting specific perturbations in metabolism, but more research needs to be done to determine the causes of the changes in amino acid levels, the relationship of these changes to decreased glucose metabolism, and the effects these changes have on the brain. Examining the activity of more enzymes involved in amino acid metabolism and building models of amino acid metabolism could help explain the alterations in amino acid levels in AD brain.

### 4.4. Glutamine

Glutamine is the most prevalent amino acid in plasma, and glutamine and glutamate are the most prevalent amino acids in human brain [71]. Glutamine supplementation decreases tau phosphorylation and has shown other protective effects such as decreasing inflammation in a mouse model of AD [72]. Glutamine levels decline in AD patient brain causing a compensatory increased expression of glutamine synthetase in some neuronal populations. It has been found that glutamine, glutamate, aspartate, alanine, and purines are likely degraded as the top alternative energy sources in neurodegenerative diseases such as AD when glucose metabolism is disturbed [73]. These amino acids are readily broken down because high levels of aminotransferases for the initial step in the catabolism of alanine, aspartate, and glutamate are present in brain [71]. In one study of AD patients, glutamine levels were found to decline in the serum [21] but increase in the temporal cortex of the brain [22]. Glutamine and alanine levels have also been found to be decreased in the blood of patients with transient global amnesia [74].

There has not been much data generated on how the brain maintains balances of amino acids and total nitrogen levels during times of neuronal amino acid catabolism, but it is likely that BCAAs can be taken up through the blood-brain barrier (BBB) and glutamate can be released to maintain nitrogen balance [75]. BCAA-derived carbons can then be fed into the citric acid cycle to form alpha-ketoglutarate, and then the alpha-ketoglutarate can be transaminated to glutamate to maintain glutamate levels. During times when ammonia levels slowly increase in the brain, exporting a glutamine (containing two nitrogen atoms) from the brain for every BCAA (or another amino acid containing a single nitrogen atom) taken up would allow for a net efflux of nitrogen to lower the brain ammonia levels [71]. However, this mechanism of removal of nitrogen is likely not robust enough to deal with large increases in brain ammonia that are known to cause encephalopathy.

### 4.5. Sulfur-Containing Amino Acids

The sulfur-containing amino acids are cysteine and methionine. Much research has been performed on supplementation with cysteine and the more membrane permeable form N-acetylcysteine (NAC) as cysteine levels limit the synthesis of glutathione, one of the most important antioxidants in the body. Cysteine and NAC cross the blood-brain barrier slowly, so other therapies are under development to increase brain glutathione levels [76]. There have been 3 small clinical trials of NAC supplementation to AD patients with mixed results [77]. Therefore, more research is needed to clarify the effects of NAC on AD. There seems to be a disruption in sulfur-containing amino acid metabolism in AD patients as serum and brain homocysteine levels increase [26]. Cysteine levels were also shown to be increased in the hippocampus of autopsied AD patients [15]. Homocysteine-cysteine disulfide levels were found to increase in AD patient serum while methionine levels decreased [27]. Increased homocysteine levels in the plasma are a known risk factor for AD and other dementias [78]. Dietary methionine supplementation caused increased amyloid-beta and phosphorylated tau levels in brain and cognitive impairment in wild-type mice [30]. Dietary methionine restriction led to decreased amyloid-beta levels and neuroprotection in APP-PS1 AD mice [79] and decreased mitochondrial complex I-mediated superoxide production and increased lifespan in rats [80], while cysteine supplementation led to a slight decrease in mTOR activity [29]. However, cysteine supplementation prevented the decreased ROS production in methionine-restricted animals.

## 5. Amino Acids as an Energy Source in AD Neurons

One of the hallmarks of AD is dysfunctional energy metabolism. Mitochondrial-derived oxygen free radicals produced in AD brain are known to damage glycolytic enzymes such as enolase [81] and glyceraldehyde-3-phosphate dehydrogenase [82], slowing glycolysis. This damage, combined with decreased insulin signaling in AD brain [83], results in decreased glucose uptake and metabolism which has been confirmed as an early event in AD progression through the use of fluorodeoxyglucose (FDG)-PET scans [84]. The decreased glucose metabolism results in decreased pyruvate production which, combined with amyloid-beta-mediated mitochondrial complex IV inhibition [85] and tau-mediated mitochondrial complex I inhibition [86], results in decreased mitochondrial energy metabolism and ATP levels. Most cells in the body show metabolic flexibility and can increase mitochondrial fatty acid beta-oxidation to maintain cellular ATP levels when glycolytic output decreases. Neurons contain very low levels of fatty acid beta-oxidation enzymes [87], so they instead rely upon ketone body catabolism, amino acid catabolism, or catabolism of lactate released from astrocytes [88] to maintain cellular ATP levels. Ketone body levels are normally very low in the well-fed and unexercised human body [89]. Therefore, amino acid catabolism, together with lactate metabolism and limited glucose metabolism, likely plays an essential role in maintaining cellular ATP levels in AD neurons. Data supporting this hypothesis come from clinical studies that show a 44% decrease in glucose utilization in autopsied early-onset familial AD brain. Surprisingly, the AD brains showed no deficits in oxygen utilization as free amino acids (and perhaps lactate) were oxidized for energy generation in replacement of glucose, leading to decreased amino acid levels [90]. Once brain amino acid levels were depleted, brain ammonia levels decreased as well. These data indicate that amino acid supplementation or high protein diets may help to energize the AD brain.

The most abundant amino acids present in the human brain as potential energy sources are glutamate and glutamine, present at roughly 7-8 mM, while the next most abundant amino acids are aspartate and taurine which are present at roughly 1.2 mM and then serine, GABA, and glycine which are present at roughly 0.5 mM [71]. The brain contains a glutamate-glutamine cycle where glutamate is released by neurons into synaptic clefts; the glutamate is then taken up by astrocytes where some is broken down for energy, but most is converted to glutamine which is exported from the astrocytes and taken up once again by neurons and converted back into glutamate. To facilitate this cycle, neurons possess high levels of two glutaminase genes,* GLS1* and* GLS2* (phosphate-activated mitochondrial glutaminase), to function in the breakdown of glutamine to glutamate, a process that releases ammonia, but small amounts of glutaminase (mostly GLS1) have also been localized to astrocytes [91]. GLS2 activity is strongly upregulated by ADP [92] and has been shown to decline with aging [93], particularly in AD brain [94].

Further release of ammonia in the brain can occur if glutamate is catabolized to alpha-ketoglutarate by glutamate dehydrogenase. In addition to the normal mammalian glutamate dehydrogenase gene,* GLUD1*, primates have a second glutamate dehydrogenase gene,* GLUD2*, that is expressed in astrocytes and may be needed in those cells to preserve alpha-ketoglutarate levels in the presence of high glutamine synthetase activity [95]. Glutamine synthetase activity has been shown to decline in AD brain likely through oxidative inactivation [96], although protein levels have been shown to increase in the prefrontal cortex and CSF. Mitochondrial GLUD1 is negatively regulated by the GTP formed from citric acid cycle function, while GLUD2 is relatively unaffected by guanine nucleotides but is positively regulated by ADP and branched chain amino acids [97]. The negative regulation of GLUD1 when energy levels are high allows for preservation of glutamate levels needed for neurotransmission as well as preventing the toxic buildup of ammonia. Glutamate can also be metabolized to alpha-ketoglutarate to fuel citric acid cycle metabolism without the release of free ammonia through the function of the alanine, aspartate, and branched chain aminotransferases [98] if the corresponding ketoacids are present in adequate amounts.

Normally both neurons and astrocytes oxidize glucose by glycolysis and the resulting pyruvate by oxidative phosphorylation to maximize ATP yield [99]. However, there is much evidence that indicates astrocytes are more metabolically flexible than neurons due to their slightly lower energy demands [100]. Therefore, astrocytes can survive predominately by glycolysis with little oxidative metabolism. In metabolically stressful times, such as in AD, astrocytes may convert the pyruvate produced from glycolysis into lactate to maintain the cellular redox state and then export the lactate from the cell. The lactate is then taken up by neurons, converted back into pyruvate, and used for oxidative energy metabolism, a process called the astrocyte-neuron lactate shuttle [101]. Astrocytes may also be able to increase the amount of glutamine released to neurons to be utilized as an energy source under the pathological conditions when neuronal glycolysis is impaired. Astrocytes possess some capability for fatty acid beta-oxidation [87], which likely becomes more important during these pathological conditions.

A study using AD mice found that a high protein/low carbohydrate diet resulted in a 5% reduction in the brain weight in AD mice, including decreased neuronal density and volume in the CA3 region of the hippocampus that is important for memory [102]. A high protein/low carbohydrate diet has also been associated with increased excitotoxicity in the aged brain [103]. These data suggest that high levels of amino acids or products of their catabolism may contribute to neurodegeneration. Consistent with this assessment, catabolism of branched chain ketoacids by branched chain ketoacid dehydrogenase (BCKDH) in mitochondria results in substantial production of damaging superoxide radical [104]. In addition, mice on a low protein/high carbohydrate diet lived longer than those on a high protein/low carbohydrate diet and had lower insulin levels and lower mTOR activation [105]. However, the health benefits of a low protein/high carbohydrate diet may only extend through middle age, as aged mice or elderly human subjects on a high protein diet showed protection from disease [106]. Therefore amino acid supplementation therapies may best be explored as therapies for late-onset AD. Plasma levels of all three branched chain amino acids showed a positive correlation with dietary protein intake in mice, while the plasma levels of most other amino acids showed a negative correlation with dietary protein intake. Therefore, some of the beneficial health effects conferred by the low protein diet in young and middle aged mice may be mediated by decreased plasma branched chain amino acid levels.

## 6. The Urea Cycle and AD

### 6.1. Amino Acid Metabolism, Ammonia, and the Urea Cycle

Proteins are digested in the stomach and intestine by several different peptidases into free amino acids and dipeptides; the dipeptides are further catabolized into amino acids by first pass hepatic metabolism. These amino acids are then either catabolized and used as substrates for gluconeogenesis in the liver or transported by the blood to other tissues where the amino acids are used for protein synthesis or broken down in processes that produce ammonia when levels exceed their requirements. For this to occur the *α*-amino group of the amino acid is often transferred to *α*-ketoglutarate to form glutamate and an *α*-ketoacid, which is oxidized for energy generation. Glutamate can undergo oxidative deamination to form ammonia and *α*-ketoglutarate, or the amino group can be transferred to oxaloacetate to form aspartate and *α*-ketoglutarate. Aspartate is required for urea cycle function in the liver. In the brain and muscle (tissues normally lacking appreciable urea cycle function) aspartate can enter the purine nucleotide cycle to release fumarate and ammonia. Other reactions produce ammonia as well; histidine, serine, threonine, and catecholamine (tyrosine-derived) catabolism release ammonia through separate reactions. Ammonia is toxic and needs to be eliminated quickly or converted to a less toxic form. In peripheral tissues once ammonia and glutamate combine to form glutamine through the action of the glutamine synthetase enzyme, the glutamine is exported from the tissue and transported through the blood to the liver where the free ammonia is released through the action of the glutaminase enzyme. The urea cycle then functions to convert the ammonia to urea, which is excreted from the body.

**Figure 1:**
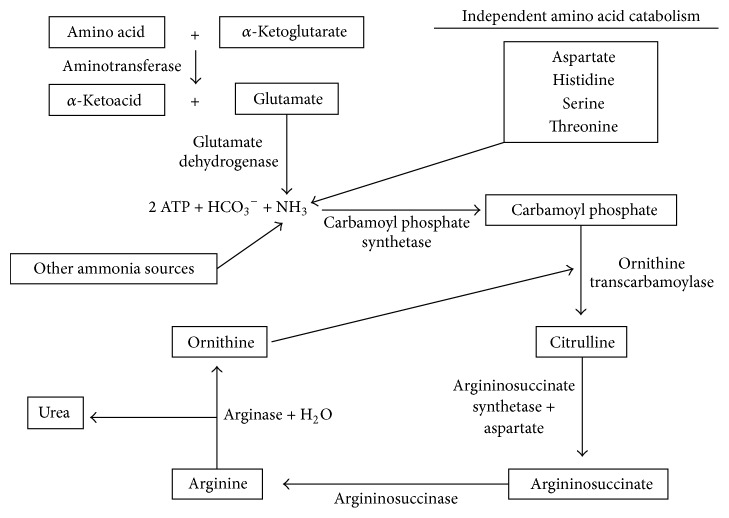
Overview of the urea cycle. The ammonia that is produced by amino acid catabolism is converted into urea in the urea cycle for excretion. The metabolic intermediates in the figure are placed in boxes and the enzymes and other necessary substrates are present next to the arrows. The levels of several of these metabolic intermediates are altered in the brain and plasma of Alzheimer's disease patients.

The first and second steps of the urea cycle occur in the mitochondria, while the other three steps occur in the cytoplasm. First, ammonia combines with ATP and HCO_3_^−^ to form carbamoyl phosphate. N-Acetylglutamate is required as a cofactor for this reaction to proceed. Carbamoyl phosphate reacts with ornithine to produce citrulline, which is transported out of mitochondria and then reacts with aspartate to form argininosuccinate. Argininosuccinate is converted by argininosuccinase into fumarate and arginine. In the final step, arginase converts arginine into ornithine and urea.  Figure 1 summarizes amino acid catabolism leading up to and including the urea cycle.

### 6.2. Changes in Components of the Urea Cycle in AD

Levels of enzymes and metabolic intermediates of the urea cycle are altered in patients with AD. All enzymes required for the urea cycle are expressed in liver, with low level urea cycle activity also occurring in the kidneys and intestines [107]. It has been shown that normal human brain has very low or no ornithine transcarbamoylase (OTC) activity, thus preventing urea cycle activity [108]. Carbamoyl phosphate synthetase activity is also low in brain tissue. However, studies using autopsied brains from AD patients have challenged this exclusive localization of the urea cycle. Hansmannel and colleagues identified mRNA expression for all enzymes of the urea cycle in the brains of both normal adults and patients with AD [109]. However, the mRNA levels of OTC were extremely low in the non-AD subjects, and the normal cytoplasmic urea cycle enzyme arginase 1 (ARG1) was extremely low in both populations. Arginase is one of the better studied urea cycle enzymes with expression that appears to be dysregulated in AD. Arginase converts arginine to urea and ornithine (see Figure 1). Two groups, Lui et al. [5] and Hansmannel et al. [109], found the same trend of increased mitochondrial arginase II (ARG2) levels in autopsied AD patient brain. Hansmannel et al. used RT-PCR to find a 55% increase in ARG2 mRNA levels in AD patients compared to controls [109], whereas Lui et al. used Western blot to show an increase in the total amount of ARG2 protein in two different brain regions with no change in a third [5].

There are several important consequences of increased* ARG2* expression in AD brain. First, increased arginase activity would likely increase urea and ornithine levels, the latter being a precursor of polyamine synthesis. Polyamines can play an important neuroprotective role in the brain. Second, increased arginase activity would likely decrease arginine levels, which can lead to decreased mTOR activity. Arginine is also a substrate for nitric oxide synthase which produces the vasorelaxing free radical nitric oxide that can increase neuroinflammation. Therefore, transgenic overexpression of ARG1 showed neuroprotection in a tau-overexpressing model of AD [110]. However, an arginase inhibitor showed neuroprotective effects in an amyloid-beta-producing mouse model of AD [111]. Therefore, it is possible that arginase expression has different effects on amyloid and tau pathology. ARG2 is the main isoform in AD brain and is highly expressed in endothelial cells. Therefore, it is also possible that ARG1 activity is neuroprotective while ARG2 activity is neurotoxic due to expression in different cell types or different subcellular localizations.

Bensemain et al. used RT-PCR to detect the transcription of the ornithine transcarbamylase (*OTC*) gene and other enzymes of the urea cycle in AD brains as well [108]. OTC activity was exclusively localized to brain endothelial cells, and its activity in cerebrospinal fluid was nearly 9 times higher in AD patients than in the control group [108]. It is interesting that OTC activity was concentrated in the endothelia in the vasculature of the brain in AD [108]; these areas are severely affected by amyloid plaques [112]. Taken together, these results indicate that the urea cycle may occur in the endothelial cells of AD patients, but this may rely upon the transport of arginine from the cytoplasm to the mitochondria to be metabolized by ARG2. The mitochondrial ornithine carriers ORC1, ORC2, and SLC25A29 are also able to transport arginine [113]. ORC1 and ORC2 are expressed at very low levels in brain [114], but this may be enough to allow low level urea cycle activity in the endothelial cells from AD patients.

Perhaps the most notable urea cycle metabolite change in the AD brain is in the level of urea itself. The direction of the change in level of urea depends on the clinical or pathological sample or the mouse model tested. Serum from human AD patients showed a 44% decrease in urea levels when assayed using GC/MS [14]. The same group found decreased urea in the serum of APP/PS1 AD model mice [21]. A decrease in urea in the hippocampus of the senescence-accelerated SAMP8 mice was also measured [115]. SAMP8 mice show neurodegeneration similar to that observed in AD. The decreased urea levels are consistent with decreased arginase levels found in APP/PS1 mouse brain [30]. Studies of human brain show markedly different results. A study by Gueli and Taibi using GC/MS on temporal lobe extracts demonstrated that urea was increased in brain tissue of AD patients over 2-fold [22]. Xu and colleagues measured urea in six different regions of the brain to find that urea was increased in AD patients' brains by an average of more than 5-fold [15]. This increase in urea levels is consistent with the increased ARG2 levels in human AD brain. Interestingly, in the striatum of postmortem Huntington's disease brain, urea was found to be the most downregulated (3.2-fold) metabolite [116], but another study found opposite results that urea was upregulated in all brain regions examined in postmortem Huntington's patients [117].

Ornithine levels were decreased in AD brain and serum [5, 14, 15]. Although ornithine is the product of an enzyme that is upregulated (ARG2), the decrease is consistent with other findings because ornithine is the substrate of OTC, another upregulated enzyme in AD brain [108], and ornithine is a precursor for the production of polyamines. Consistent with this reasoning, the level of the polyamine spermidine was found to increase by 70% in the temporal cortex of AD brain. [118]. Citrulline levels, however, remain unchanged in AD brains [5, 119]. Citrulline is a strong antioxidant and citrulline supplementation prevented age-related changes in lipid metabolism in mouse hippocampus [120]. Aspartate reacts with citrulline to form argininosuccinate. Aspartate levels are decreased in AD patient serum [14], and both aspartate and arginine levels are decreased in the brain of AD patients [15, 22]. Decreased levels of urea cycle intermediates could indicate their efficient metabolism. Considering that different groups have shown increased urea levels in autopsied AD brains as well as increased expression of one or more urea cycle genes, current evidence suggests that urea cycle activity may be induced in endothelial cells from AD patient brain. It is possible that a urea cycle metabolite such as arginine that is produced in neurons and glia is imported into AD endothelial cells where ARG2 levels are high and OTC is exclusively present to finish the urea cycle there. The citrulline produced from endothelial cell OTC activity could also be exported to neurons or glia to finish the urea cycle. However, it is also possible that the higher urea levels found in AD brain are strictly due to increased ARG2 levels independent of complete urea cycle function.

Increased urea levels in AD brain raise questions as to what could be leading to the increased expression of ARG2 (and OTC). The main function of the urea cycle is to process nitrogenous waste produced from amino acid catabolism and other sources into a less toxic form before removal from the body. Therefore, it has been hypothesized that abnormal nitrogen metabolism may play a role in the pathogenesis of AD [121]. One of the early hypotheses for the pathogenesis of AD, proposed by Seiler in 1993, was the ammonia hypothesis; this posits that increased levels of ammonia accumulate in and are toxic to the AD brain [122]. However, the amyloid cascade hypothesis was proposed the year before [123], and the ammonia hypothesis was not thoroughly investigated [121]. The ammonia hypothesis of AD was generated due to the following observations: increased ammonia levels measured in the plasma from AD patients [124, 125], decreased glutamine synthetase enzyme activity in AD astrocytes to scavenge ammonia [96, 126], increased adenosine deaminase activity in AD brain [127], and increased monoamine oxidase activity in AD brain [128, 129] (the latter two enzymes produce ammonia). Ammonia has also been implicated as a cause of oxidative damage in the brain because it was found to increase reactive oxygen species levels in SH-SY5Y cells [130] and astrocytes [131] and lead to RNA oxidation in rats [132].

Furthermore, mitochondrial activity in rat and mouse models is impaired by ammonia. Ammonia toxicity in rodent brains led to decreased state III respiration [133] and decreased cytochrome c oxidase (complex IV) activity [134], as well as decreased activity of several other enzymes in isolated synaptic mitochondria [135]. Impaired mitochondrial function is often associated with increased oxidative damage. This may in part explain the increase in reactive oxygen species in the presence of ammonia. Increased ammonia production would either necessitate urea cycle function to metabolize the toxic ammonia to urea or necessitate increased reaction of ammonia with glutamate catalyzed by glutamine synthetase followed by export of glutamine from the brain. Evidence from studies of the urea cycle and amino acid metabolism in AD subjects and mouse models justifies further investigation of the regulation of the production and detoxification of ammonia in the AD brain.

## 7. Considerations for Dietary Metabolite Supplementation as a Treatment for AD

Increasing or decreasing the levels of specific amino acids and other metabolites in the diet has shown some promise for improving markers of aging and longevity [41]; so it is possible that nutrient supplementation or restriction may improve neural functioning in AD patients because age is the major risk factor for AD. However, there are several hurdles to overcome before an efficacious treatment can be formulated. For example, studies on intestinal transport, bioavailability, hepatic metabolism and excretion, and blood-brain barrier transport are needed in order to choose the optimal formulations. Much of this information is present for a few commonly studied amino acids, but much of it is absent for the majority of the amino acids. From what is known, it appears that hepatic metabolism presents a large challenge to overcome for the supplementation of many of the amino acids for their use in the treatment of neurodegeneration, but intestinal transport may also become limiting in the elderly [106]. Several of the amino acids also have limited blood-brain barrier permeability. We will present one promising strategy below taking these many challenges into consideration.

As mentioned previously, intestinal uptake of amino acids declines past age 65. It has been shown that the bioavailability of individual amino acids and dipeptides is slightly better than that of amino acids consumed as polypeptides since the individual monomers can be absorbed quickly without the need for further enzymatic hydrolysis in the gut. Therefore, dietary supplementation with individual amino acids or combinations of individual amino acids would likely benefit the elderly in addition to a high protein diet that promotes health in this age group [106]. The use of individual amino acids also has the added benefit of being able to stimulate specific signaling pathways. Due to the decreased intestinal uptake of amino acids in the elderly, they may particularly benefit from supplementation with hydrophobic, more membrane permeable forms of amino acids such as amino acid ethyl esters or N-acetyl amino acids. These amino acid derivatives show a greater probability of diffusion across membrane bilayers such as intestinal epithelia and the capillary endothelia of the blood-brain barrier where the activity of specific membrane transporters may be limiting. These more hydrophobic amino acid derivatives are cleaved by esterases and other hydrolytic enzymes intracellularly or extracellularly to release the free amino acid. This hydrolysis may occur to a large extent during first pass hepatic metabolism, so this strategy may be of marginal use for increasing the blood-brain barrier permeability for many amino acids.

Dietary aspartate, glutamate, and glutamine are oxidized as primary sources of fuel for intestinal cells [136]. In addition, glutamate and aspartate are transported very poorly through the blood-brain barrier [137], even though they are present at high concentrations in the brain. Other amino acids that are transported poorly through the blood-brain barrier include glycine, alanine, proline, and GABA. Medium and large side chain, nonpolar amino acids are transported relatively well by the blood-brain barrier into the brain, including aromatic amino acids, BCAAs, methionine, histidine, and threonine [138]. Glutamine and asparagine are also likely transported by the same pathway. These amino acids all compete for transport through the L1 transport pathway. Another amino acid transporter called the y^+^ system transports basic amino acids such as arginine, lysine, and ornithine as well as several neutral amino acids such as serine through the blood-brain barrier [137]. One potential therapeutic strategy for AD patients is to supplement low protein diets with high levels of BCAAs, aromatic amino acids, glutamine, histidine, and threonine. Through competition for the L1 amino acid transport system, this therapy could limit the transport of methionine into the brain, perhaps yielding the known metabolic and neuroprotective benefits of methionine restriction [30, 44]. However, methionine is also transported into the brain to a limited extent through the y^+^ system, which may hinder the effectiveness of this strategy. A second potential therapy is to omit leucine and/or isoleucine instead of methionine from the previously mentioned supplementation strategy. These two amino acids are not transported by the y^+^ system [137]. Depletion of leucine or isoleucine in the brain could lead to amino acid imbalance, activation of GCN2 kinase, and possibly the inhibition of mTOR kinase to slow protein translation rates that may be beneficial for reducing the levels of neurofibrillary tangles formed from hyperphosphorylated aggregates of tau protein. These amino acid supplementation therapies could be combined with supplementation with other metabolic fuels such as D-beta-hydroxybutyrate (a ketone body), citric acid cycle intermediates, pyruvate, and/or lactate, which would decrease the reliance of AD neurons on the use of amino acids as a fuel. Consumption of high levels of these alternative metabolic fuels may be able to partially restore neuronal amino acid levels.

## 8. Summary and Conclusion

**Figure 2:**
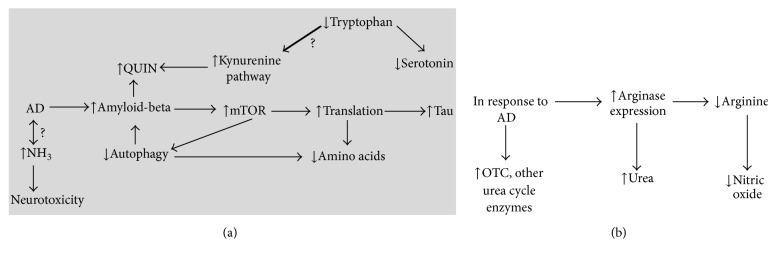
Select alterations affecting amino acid metabolism in Alzheimer's disease brain. (a) Several pathogenic processes occurring in Alzheimer's disease brain. The bold arrow indicates that tryptophan metabolism in the kynurenine pathway may be increased relative to the serotonin pathway in AD, contributing to the lower tryptophan levels observed. (b) Select mechanisms through which the Alzheimer's disease brain attempts to maintain homeostasis when faced with decreased glucose catabolism and increased amino acid catabolism and ammonia levels.

Dietary amino acids provide a large amount of carbon and nitrogen to the body that can be metabolized by a myriad of biochemical pathways. Amino acids have roles in neuronal signaling, energy production, and nitrogenous waste production and elimination. These processes are important for normal physiology, so it is not surprising that disease states result from major alterations in their function, but whether relatively minor perturbations of this metabolism contribute to neurodegeneration requires further study. Brains and serum from AD patients have shown many alterations in amino acid levels and metabolism that provide a basis for some of the symptoms of the disease. These individual changes may each play a different role in the disease, highlighting the complexity that underlies AD pathology. An increase in urea in the brains of AD patients together with the altered expression of urea cycle enzymes suggests that urea cycle activity may be induced in AD brain endothelial cells. Viewing AD as a disease with a large metabolic component provides valuable insight into possible new targets for drug discovery in the AD research field. A summary of some of the altered amino acid metabolism that occurs in AD is shown in Figure 2.

The measurement of metabolite levels provides a snapshot of a very dynamic process. While this information is extremely useful, it is not sufficient by itself to understand the pathological changes associated with AD. Further studies measuring enzyme activities could provide complementary information about the dynamics of amino acid metabolism in AD. In addition, studies overexpressing OTC and ARG2 to activate the urea cycle in the brain endothelial cells of an AD mouse model would help clarify the effects of endothelial urea cycle activity on brain physiology and cognitive function. Studying AD from a metabolic perspective could lead to dietary supplementation therapies that delay disease progression or alleviate some of the suffering caused by the disease.

## Figures and Tables

**Table 1:** Free amino acid abundance changes in Alzheimer's disease.

Amino acid	Tissue or fluid measured	Increase or decrease in AD	Points of interest	Effects of addition	DataWarrior Drug Score^*∗*^
Leucine			Strong mTOR activator [10]; *α*-ketoacid metabolite led to metabolic dysfunction [10–12]	Metabolite led to mitochondrial dysfunction in rat neurons [12]; BCAAs increased mean lifespan of male mice [13]	0.592
Valine	Plasma [14]	Decrease [14]	mTOR activator [10]; *α*-ketoacid metabolite led to oxidative stress [10]	BCAA-fortified diet increased mean lifespan of male mice [13]	0.559
Isoleucine			mTOR activator [10]; *α*-ketoacid metabolite led to oxidative stress [10]	BCAA-fortified diet increased mean lifespan of male mice [13]	0.684
Phenylalanine	Serum [14]; brain [15, 16]	Decrease [14]; increase [15, 16]	Metabolized in the absence of tyrosine [17]		0.579
Tryptophan	Serum [14]; brain [15]	Decrease [14]; increase [15]	Metabolite causes metabolic dysfunction [18]	Metabolite increases nitric oxide synthetase in cell culture [19]	0.661
Tyrosine	Serum [14]	Decrease [14]	Decrease levels could disrupt catecholamine production	Improved memory and cognitive function in humans [20]	0.584
Glutamine	Serum [21]; brain [22]	Decrease [21]; increase [22]			0.573
Aspartic acid	Brain [15, 22]; serum [14]	Decrease [14, 15, 22]			0.593
Glutamic acid	Serum [23]; brain [15, 22]	Increase [15]; decrease [22, 23]	Excitotoxicity leads to neuronal death [24, 25]		0.531
Lysine	Brain [15]	Decrease [15]			0.499
Histidine	Serum [14]	Decrease [14]			0.835
Cysteine	Serum [26, 27]; brain [15, 26]; CSF [28]	Decrease [26]; increase [15, 27, 28]	Involved in glutathione synthesis; mTOR inhibitor	Decreased mTOR activity in rats [29]	0.493
Methionine	Serum [27]	Decrease [27]	mTOR activator	Increased amyloid-beta and p-tau in mice [30]	0.578

^*∗*^Structures were drawn using DataWarrior software using their most prevalent charge states at pH 7.4. A higher drug score value indicates a better drug candidate.
